# Rare Cause of Inguinal Pain in 39-year-old Male

**DOI:** 10.5811/cpcem.2019.1.41212

**Published:** 2019-03-05

**Authors:** Kieron Barkataki, Nathan Wang, Daniel Quesada, Rachell O’Donnell, James W. Rosbrugh, Phillip Aguìñiga-Navarrete

**Affiliations:** * Kern Medical, Department of Emergency Medicine, Bakersfield, California; † LAC+USC Medical Center, Department of Emergency Medicine, Los Angeles, California

## CASE PRESENTATION

A 39-year-old male with no past history presented with three months of left inguinal pain and a left groin lump that became progressively larger and more painful. He was seen at another hospital over one month prior where they were unable to manually reduce the lump. He could not recall the computed tomography (CT) findings, and no surgery was performed. Since then, he has experienced persistent left inguinal pain and nausea. He denied fever, vomiting, dysuria, hematuria, penile discharge, testicular pain, or history of sexually transmitted diseases.

**Image 1:**
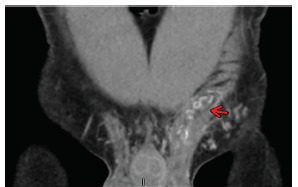
Coronal contrast-enhanced computed tomography of the abdomen and pelvis showing fullness in the left inguinal canal with surrounding fat stranding (arrow).

**Image 2:**
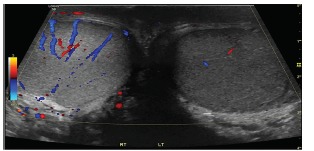
Formal ultrasound of both testes with demonstration of decreased Doppler vascular flow on left testicle compared to right.

**Image 3:**
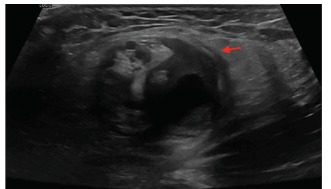
Transverse view of formal ultrasound of the left inguinal canal displaying prominent heterogeneous structures (arrow).

Physical exam revealed a firm, tender, and non-reducible mass in the left inguinal canal and along the spermatic cord. Remainder of examination was normal. Complete blood count, basic metabolic panel, lactate, urinalysis and urine culture were normal. CT of the abdomen and pelvis was suggestive of pampiniform plexus thrombosis (Image 1). Formal ultrasound images revealed diminished Doppler vascular flow (Image 2) within the left testicle and prominent, heterogeneous vascular structures seen in the left inguinal canal (Image 3) that correlated with the CT, indicating pampiniform plexus thrombosis as well.

## DISCUSSION

Pampiniform plexus thrombosis is a rare cause of inguinal pain, which is often misdiagnosed as hernia or orchitis. Accurate diagnosis can prevent unnecessary treatment including surgical intervention. There are limited references in the literature to this condition, and there are no evidence-based approaches to management. The majority of reported cases involved the left venous plexus, and most were diagnosed intra-operatively for pre-operative diagnoses of incarcerated inguinal hernia or orchitis.^1^ A work-up for hypercoagulability is recommended.^2^ Management has ranged from conservative treatment with nonsteroidal anti-inflammatory drugs to surgical excision of the thrombosed vessels.^3^

**CPC-EM Capsule**

What do we already know about this clinical entity?

*Pampinifom plexus thrombosis is a rare cause of inguinal pain. There is little consensus about the appropriate treatment at this time*.

What is the major impact of the image(s)?

*This disease process is often mistaken for incarcerated inguinal hernia. Multiple cases were not accurately diagnosed until the patient was on the operating table*.

How might this improve emergency medicine practice?

*This case reminds physicians of rare clinical entities and may help avoid unnecessary surgery*.
